# Gut Microbiota and Mucin Composition in Female Broiler Chickens Fed Diets including Yellow Mealworm (*Tenebrio molitor*, L.)

**DOI:** 10.3390/ani9050213

**Published:** 2019-05-03

**Authors:** Ilaria Biasato, Ilario Ferrocino, Elena Grego, Sihem Dabbou, Francesco Gai, Laura Gasco, Luca Cocolin, Maria Teresa Capucchio, Achille Schiavone

**Affiliations:** 1 Department of Agricultural, Forest and Food Sciences, University of Turin, Largo Paolo Braccini 2, 10095 Grugliasco (TO), Italy; ilaria.biasato@unito.it (I.B.); ilario.ferrocino@unito.it (I.F.); laura.gasco@unito.it (L.G.); lucasimone.cocolin@unito.it (L.C.); 2 Department of Veterinary Sciences, University of Turin, Largo Paolo Braccini 2, 10095 Grugliasco (TO), Italy; elena.grego@unito.it (E.G.); sihem.dabbou@unito.it (S.D.); achille.schiavone@unito.it (A.S.); 3 Institute of Science of Food Production, National Research Council, Largo Paolo Braccini 2, 10095 Grugliasco (TO), Italy; francesco.gai@ispa.cnr.it

**Keywords:** insect meal, microbiota, mucin, insect meal

## Abstract

**Simple Summary:**

Gut health evaluation is a topic of great research interest in animal production, since the intestinal features (such as the microbiota and the mucin composition, as well as the mucosal morphology) are usually diet dependent, thus also directly influencing the growth performance of the animals. Insects are currently considered a novel, promising alternative protein source for animal feeding due to their remarkable nutritional properties, low competitiveness with human food and environmental implications, but data regarding the gut health of insect-fed animals are still very limited. We herein demonstrated that yellow mealworm (*Tenebrio molitor*, TM) meal utilization at low inclusion rates (5%) represents the most feasible alternative in terms of gut microbiota characteristics (identification of a phylum profile with better feed digestion and higher capacity of harvesting) and mucin dynamics (higher mucin production) in broiler chickens.

**Abstract:**

A total of 160 female broiler chickens were divided into four dietary treatments (control feed [C] and 5, 10 and 15% TM meal inclusion, respectively, with five replicate pens/treatment and eight birds/pen) to investigate the effects of *Tenebrio molitor* (TM) meal utilization on poultry gut microbiota and mucin composition. The cecal microbiota assessment displayed a shift in the beta diversity in chickens fed TM-based diets. The TM10 and TM15 birds showed a significant decrease in the relative abundance of *Firmicutes* phylum and lower *Firmicutes*:*Bacteroidetes* ratios (False Discovery Rate [FDR] < 0.05), respectively, than the TM5 group. The relative abundance of *Clostridium*, *Alistipes* and *Sutterella* genera significantly increased in TM chickens (FDR < 0.05), while birds fed TM-based diets displayed a significant decrease in the relative abundance of *Ruminococcus* genus in comparison with the C group (FDR < 0.05). Gut mucin composition evaluation revealed higher mucin staining intensity in the intestinal villi of TM5 birds than the other TM groups, as well as mucin reduction in the intestinal villi of TM10 birds when compared to the C group (*p* < 0.05). In conclusion, dietary TM meal utilization (especially the 10–15% inclusion levels) may negatively influence either the cecal microbiota or the intestinal mucin dynamics of broiler chickens.

## 1. Introduction

Effective functionality and health of the gastrointestinal tract (GIT) are important factors in determining animal performance [1]. These aspects are particularly relevant in poultry farming, where animals capable of growing rapidly within a short period of time are needed.

Three components of gut health have previously been suggested: diet, mucosa (which is, in turn, characterized by the digestive epithelium, the gut-associated lymphoid tissue and the mucus layer), and commensal flora [1,2]. All these components widely interact, establishing a delicate and dynamic equilibrium within the GIT environment that guarantees the proper and efficient functionality of the digestive system and, as a consequence, maintenance of animal health, welfare (animal behavior included) and performance [1]. In particular, an extensive, two-way communication exists between gut microbiota and the mucosal barrier. On the one hand, gut microbiota contribute to several physiological (i.e., development and maturation of the immune system) and metabolic (i.e., fermentation of the non-digestible dietary components, modulation of endogenous epithelial-derived mucus secretion, regulation of intestinal epithelial cell differentiation and proliferation) functions of the GIT mucosal barrier. On the other hand, the GIT mucosal immune system can provide the microbiota with several substances such as mucus and antimicrobial peptides in order to protect the host against the invasion of bacteria through the intestinal walls [1]. Among the GIT-produced substances, the mucus, an adherent gel layer that covers the entire surface of the GIT mucosa, represents the first barrier between the intestinal lumen and the absorptive cells. It is implicated in several physiological processes, since it acts as a lubricant enhancing the propulsion of chyme, modulates nutrient digestion and absorption because of its permeability, protects the underlying epithelium from physical and chemical injury and prevents the entry of enteric pathogens [3]. Mucins, the main component of the mucus layer and the principal determinants of their key properties, are highly glycosylated glycoproteins, which are synthesized, stored and secreted by goblet cells of the GIT epithelium [3]. Their protein backbone is glycosylated by carbohydrate chains composed of different monosaccharides whose chemical nature allows mucin histological differentiation into two broad categories: neutral and acidic, with the latter being further subdivided into sialylated and sulfated mucin types [4]. Bacterial colonization and proliferation have been reported to widely influence gut mucin composition, in particular by the synthesis of mucin-specific glycosidases, glycosulfatases and proteases [5,6].

Diet can perfectly fit within the complex relationship intervening between gut microbiota and the mucosal barrier, since the ingested nutrients may remarkably influence both the development and the functionality of the GIT [2]. Indeed, dietary nutrients may alter gut microbiota composition and its functions by modulating the synthesis of antimicrobial peptides or other metabolites that have a direct influence on either the growth or the adhesion of specific pathogens to the intestinal mucosa. Furthermore, diet is capable of directly modifying the GIT epithelium by controlling cytokine production and influencing mucosal barrier functions and characteristics [1].

Insects—whose role as feed ingredients in animal nutrition has widely been investigated—may also represent remarkable sources of valuable compounds (i.e., chitin, lauric acid, and antimicrobial peptides) capable of exerting positive effects on gut health and the overall health status of animals [7]. To date, the implications of insect meal utilization on poultry gut health have been investigated in *Tenebrio molitor* (TM)-fed free-range chickens [8,9] and TM- [10,11] and *Hermetia illucens* (HI)-fed broiler chickens [12] by the evaluation of intestinal microbiota, morphology and mucin composition together [9] or intestinal morphology alone [10,11,12]. Furthermore, Borrelli et al. [13] recently investigated the effects of HI larva meal administration on cecal microbiota and short-chain fatty acid (SCFA) production in laying hens. However, studies about the influence of insect meal utilization on the gut microbiota and mucin composition of broilers are still lacking.

Based on the above reported background, the current research aims to evaluate the effects of dietary TM meal inclusion on the gut microbiota and mucin composition of female broiler chickens.

## 2. Materials and Methods

### 2.1. Birds and Experimental Design

The experimental design of the present study is reported by Biasato et al. [10]. The experimental protocol followed the guidelines of the European and the Italian laws regarding the experimental animals (European Directive 86 609/EEC-Italian law D.L. 116/92), and was also approved by the Ethical Committee of the Department of Veterinary Sciences of the University of Turin (Ref. 4, 23/06/2014). In order to give a brief summary, 160 1-day-old female broiler chicks (Ross 708) were randomly distributed to four dietary treatments. Each diet was offered to 5 replicates (pens) of 8 chicks. Corn meal-, corn gluten meal-, and soybean meal-based diets were used as the control diets (C), while the three experimental dietary treatments (indicated as TM5, TM10 and TM15) were obtained by including 5, 10 and 15% full-fat TM larva meals (Gaobeidian Shannong Biology Co. Ltd., Gaobeidian, Hebei province-China), respectively, as partial replacements of the soybean meal, corn gluten meal and soybean oil. The chemical composition of the TM larva meal was as follows: 948 g/kg dry matter, 912 g/k organic matter, 524 g/kg crude protein, and 280 g/kg ether extract. Details of the diets are shown in Table S1). Nutrient digestibility and apparent metabolizable energy (AMEn) were previously assessed [14]. The growth performance of the broiler chickens was also evaluated throughout the experimental trial, as reported in detail by Biasato et al. [10]. Briefly, the live weight (LW), the average daily gain (ADG) and the average daily feed intake (DFI) of the birds increased with increasing levels of dietary TM meal inclusion (LW: end of the starter and the finisher periods; ADG: starter period; DFI: starter and grower periods). The feed conversion ratio (FCR) of the animals also increased during the grower period with increasing dietary TM meal inclusion levels, but the overall FCR was unaffected by insect meal utilization. The experimental period lasted 40 days.

### 2.2. Intestinal Sampling

At the end of the experimental trial, ten chickens per dietary treatment (two birds/pen) were randomly selected and slaughtered in a commercial abattoir. The birds received their last feed 12 h before slaughter. At the slaughterhouse, the animals were stunned by electrocution and exsanguinated. The cecal content was sampled using a sterilized spatula cooled at 4 °C (for a maximum of 2 h), collected into sterile plastic tubes and frozen at −80 °C until DNA extraction. Well-defined, standardized samples of both the small (duodenum, jejunum and ileum) and the large (cecum) intestine were collected and processed for histochemical staining, according to Biasato et al. [9].

### 2.3. DNA Extraction and 16S rRNA Amplicon Target Sequencing

The pooled cecal content from two chickens per pen (five pools/dietary treatment) was submitted to DNA extraction and sequencing. The DNA was extracted using a commercial kit (DNAzol^®^ Reagent, Thermo Fisher Scientific, Waltham, MA, USA) following the instructions reported by the manufacturer. The cecal microbiota was then assessed by sequencing the amplified V3–V4 region of the 16S rRNA gene through the primers and the PCR conditions previously reported [15]. Sample multiplexing, library purification and sequencing activities were carried out according to the “16S Metagenomic Sequencing Library Preparation” guide (Illumina). All the libraries were finally sequenced by BMR Genomics S.r.l. (Padova, Italy) on a MiSeq platform (Illumina Italy S.r.l., Milan, Italy), leading to 250 bp, paired-end reads.

### 2.4. Histochemical Staining

Three different histochemical staining methods were performed on the intestinal sections of ten chickens per dietary treatment (two birds/pen), according to Biasato et al. [9]: periodic-acid Schiff (for the identification of the neutral mucins), Alcian Blue pH 2.5 (for the identification of the acidic sialylated mucins) and high iron diamine (for the identification of the acidic sulfated mucins).

### 2.5. Mucin Staining Intensity Evaluation

The mucin staining intensity of goblet cells was scored semiquantitatively on one slide per histochemical staining for each intestinal segment, as reported in detail by Biasato et al. [9].

### 2.6. Bioinformatics and Statistical Analysis

Paired-end reads were first assembled by FLASH software [16] with default parameters. Quality filtering and operational taxonomic unit (OTU) clustering were performed through QIIME 1.9.0 software (Caporaso Lab, Flagstaff, AZ, USA) [17] and the recently described pipeline [18]. Alpha diversity indices were calculated using the diversity function of the *vegan* package [19] in R environment (https://www.r-project.org). In order to find the differences depending on the dietary treatment, the alpha diversity index was analyzed through the pairwise Wilcoxon rank sum test. As far as beta diversity is concerned, Weighted UniFrac distance matrices were used to identify the differences through Adonis and ANOSIM tests. The OTU table that displayed the highest taxonomy resolution was used to build the Principal Component Analysis (PCA). The Kruskal–Wallis test was used to find the differences in the OTU relative abundance according to the dietary treatment. *p*-values were adjusted for multiple testing as a false discovery rate (FDR).

The statistical analysis of the histochemical findings was performed using the IBM SPSS Statistics v. 20.0.0 software (IBM, Armonk, NY, USA). The histochemical data were analyzed using a generalized linear model (GLM) similar to those proposed by Tsirtsikos et al. [20,21] and recently adopted by Biasato et al. [9]. The results were expressed as least squares means and standard error of the mean (SEM). *p* values < 0.05 were considered statistically significant.

## 3. Results

### 3.1. Cecal Microbiota Characterization

After sequencing, 1.985.931 raw reads (2 × 250 bp) were obtained and 731.677 reads passed the filters applied through QIIME, with an average value of 36.584 reads/sample. The datasets were rarefied at 3600 reads after raw read quality filtering.

**Figure 1:**
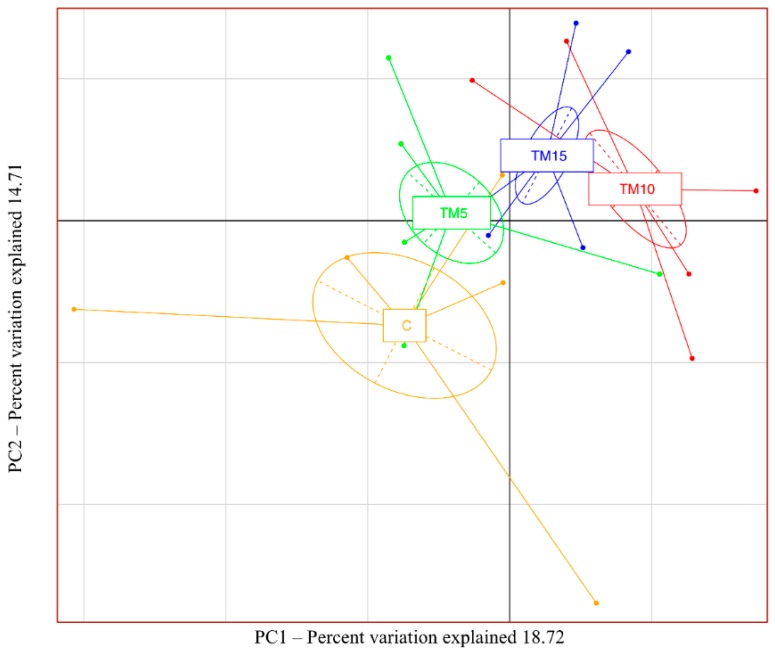
Principal Component Analysis (PCA) of the bacterial community composition in cecal samples of female broiler chickens fed with control (C), 5% (TM5), 10% (TM10) and 15% (TM15) inclusion levels of *Tenebrio molitor* meal diets.

Good’s diversity index indicated a satisfactory coverage for all the samples in both the experimental trials (average Good’s coverage of 87%, Table S2). Through alpha diversity, no significant differences between the basal and the TM-based diets were observed (*p* > 0.05, Table S2). However, significant differences among the birds fed the C and the TM-based diets were identified through Adonis and ANOSIM statistical tests based on Weighted UniFrac distance matrices (*p* < 0.001). In particular, the PCA revealed a clear and progressive separation of cecal microbiota as a function of the dietary treatment (Figure 1).

**Figure 2:**
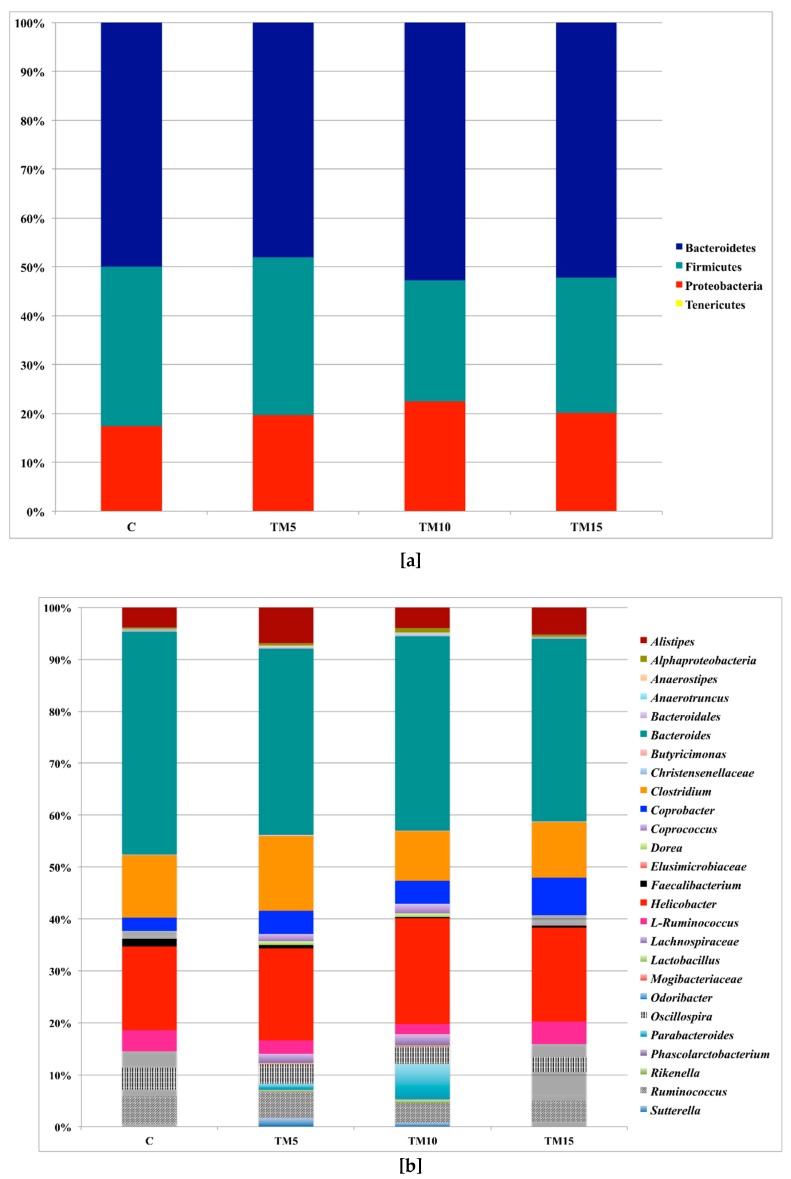
Relative abundance of the main bacterial phyla (**a**) and genera (**b**) in cecal samples of female broiler chickens fed with control (C), 5% (TM5), 10% (TM10) and 15% (TM15) inclusion levels of *Tenebrio molitor* meal diets.

Figure 2 and Table S3 summarize the relative abundances of the main phyla and genera sequenced in the cecal microbiota of the broiler chickens of the present study. In particular, *Bacteroidetes*, *Firmicutes* and *Proteobacteria* represented the three major bacterial phyla observed in both the C- and the TM-fed groups (Figure 2A, Table S3). At the genus level, ten OTUs were identified as predominant in either the birds fed the basal or the TM-based diets: *Bacteroides*, *Alistipes*, *Coprobacter* and *Parabacteroides* (within the phylum *Bacteroidetes*), *Clostridium*, *Ruminococcus*, L-*Ruminococcus* (*Ruminococcus* from the *Lachnospiraceae* family), *Oscillospira* and unclassified members (U. m.) of the *Lachnospiraceae* family (within the phylum *Firmicutes*), and *Helicobacter* (within the phylum *Proteobacteria*) (Figure 2B, Table S3).

**Figure 3:**
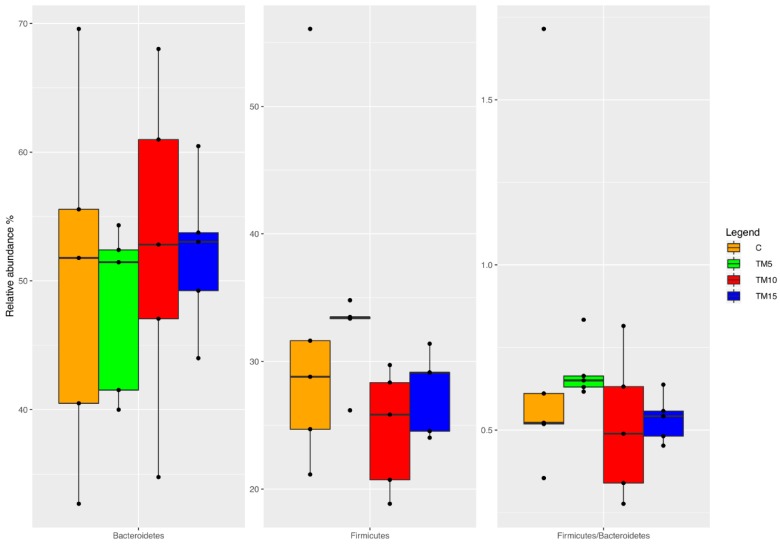
Relative abundance at the phylum level of differentially abundant operational taxonomic units (OTUs) based on Pairwise Kruskal–Wallis test (FDR < 0.05) in cecal samples of female broiler chickens fed control (C), 5% (TM5), 10% (TM10) and 15% (TM15) inclusion levels of *Tenebrio molitor* meal diets.

At the phylum level (Figure 3), the relative abundance of *Bacteroidetes* was unaffected by dietary TM meal inclusion (FDR > 0.05). On the contrary, the birds fed TM10 and TM15 diets showed a significant decrease in the relative abundance of *Firmicutes* phylum and lower *Firmicutes*:*Bacteroidetes* ratios, respectively, when compared to the TM5 group (FDR < 0.05).

**Figure 4:**
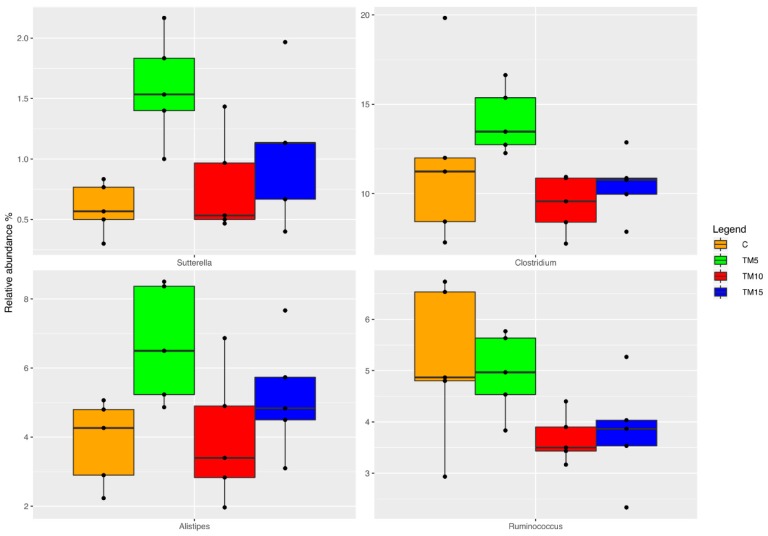
Relative abundance at the genus level of differentially abundant operational taxonomic units (OTUs) based on Pairwise Kruskal–Wallis test (FDR < 0.05) in cecal samples of female broiler chickens fed control (C), 5% (TM5), 10% (TM10) and 15% (TM15) inclusion levels of *Tenebrio molitor* meal diets.

As far as genus level is concerned (Figure 4), the relative abundance of *Sutterella*, *Clostridium* and *Alistipes* was higher in the TM- than the basal diet-fed animals (FDR < 0.05). Differently, the birds fed the TM-based diets showed a significant decrease in the relative abundance of *Ruminococcus* in comparison with the C diet (FDR < 0.05).

### 3.2. Intestinal Mucin Composition

The mucin type (*p* < 0.01), gut segment (*p* < 0.001) and crypt fragment (*p* < 0.001) significantly influenced mucin staining intensity in the intestinal crypts of the broiler chickens in the present study. On the contrary, there was no significant influence of dietary TM meal inclusion (*p* > 0.05) on the histochemical findings (Table 1). In particular, the crypts showed higher neutral mucin staining intensity (*p* < 0.01) than the other mucin types. Lower mucin staining intensity was also found in the cecal crypts (*p* < 0.001) when compared to the other gut segments, with a significant increase (*p* < 0.001) being additionally identified from the duodenum to the ileum. Furthermore, the crypt base showed greater mucin staining intensity (*p* < 0.001) than the other crypt fragments (Table 2).

Dietary TM meal inclusion (*p* < 0.01), the gut segment (*p* < 0.001) and the villus fragment (*p* < 0.05) significantly influenced mucin staining intensity in the intestinal villi, whereas there was no significant effect of the mucin type (*p* > 0.05) on the histochemical findings (Table 1). In particular, the villi of the TM5 animals showed higher mucin staining intensity (*p* < 0.01) compared to the TM10 and the TM15, but similar histochemical findings (*p* > 0.05) when compared to those of birds fed the C diet. In contrast, the villi of the TM10 animals showed lower mucin staining intensity (*p* < 0.01) compared to the C group. Furthermore, greater mucin staining intensity was observed in the jejunal and the ileal villi (*p* < 0.001) than the duodenum. The villus tip also showed lower mucin staining intensity (*p* < 0.05) than the base (Table 2).

## 4. Discussion

### 4.1. Cecal Microbiota Characterization

The present study is the first to investigate the cecal microbiota of broiler chickens fed insect-based diets. The choice of cecum as the representative gut segment was related to its characteristics: indeed, it harbors the highest microbial cell density and diversity, shows the longest digesta retention time, and represents one of the most important sites for urea recycling, water reabsorption regulation, and carbohydrate fermentation, thus positively affecting gut health and nutrition [22].

*Bacteroidetes*, *Firmicutes* and *Proteobacteria* comprised the major bacterial phyla identified in the cecal microbiota of the broiler chickens fed both the C and the TM-based diets in the present study, as previously observed in normal chickens [22,23,24,25]. However, the predominance of the phylum *Bacteroidetes* over *Firmicutes* is in contrast with these studies. The results of the current trial are more in agreement with what was observed in duck [26] goose [27] and turkey [28], where the dominant phyla, from high to low, have been reported to be *Bacteroidetes*, *Firmicutes* and *Proteobacteria*. It is well known that chickens and ducks employ roughage utilization [29] and *Bacteroidetes* members are involved in the digestion of complex polysaccharides [30]. Therefore, the above-mentioned phyla changes could reflect this poultry nutrition attitude.

Concerning the genus level, the cecal microbiota of the broiler chickens fed either the basal or the TM-based diets in the current research was mainly colonized by *Bacteroides*, *Clostridium*, *Alistipes*, *Coprobacter*, *Ruminococcus* and *Helicobacter* genera. These findings can be contextualized within the overall view of the currently available literature, where the most predominant genera found in chicken cecum have been reported to be *Clostridium*, *Ruminococcus*, *Lactobacillus*, *Bacteroides* [25,31,32,33,34] and, to a lesser extent, *Alistipes* and *Faecalibacterium* [25]. However, the percentages related to *Coprobacter* and *Helicobacter* genera particularly stand out. Firstly, identification of the *Coprobacter* genus in chicken microbiota represents an unexpected and novel finding. Indeed, this genus is highly prevalent in human intestinal microbiota and is characterized by the ability to produce propionic acid [35]. Secondly, a high abundance of the *Helicobacter* genus in chicken cecum is generally related to its potential capability of removing hydrogen, which, in turn, may benefit other GIT bacteria and help the host to recover energy from food [36]. However, another important aspect to consider is that some specific enterohepatic *Helicobacter* species (i.e., *Helicobacter pullorum*) can be detected in the gut of healthy chickens as well as in the liver and the intestine of hens with vibrionic-like liver lesions and human patients with gastroenteritis [37]. Furthermore, the identification of a high abundance of the *Helicobacter* genus cannot be excluded as a potential cause of mucin synthesis reduction in female broilers fed the 10% level of TM meal inclusion when compared to the basal diet. Indeed, bacteria such as *Helicobacter pylori* have the enzymatic ability to breakdown the oligomeric structure of the mucins, allowing the pathogens to move freely in the mucus layer, assisted by its highly active flagellum and its ability to down-regulate mucin synthesis [6].

Investigating the differences between broiler chickens fed the basal and the TM-based diets in the present study, no differences were found in regards to α-diversity measures. Concerning β-diversity, a clear separation of cecal microbiota due to dietary TM meal inclusion was, however, observed. This result is in agreement with Borrelli et al. [13] and Biasato et al. [9], who observed higher β-diversity in HI-fed laying hens and TM-fed free-range chickens, respectively, when compared to basal diet-fed birds. High levels of diversity generally help intestinal microbiota to maintain stability after environmental stress [38], as well as to determine effective colonization resistance against potential invading pathogens [39]. Therefore, the above-mentioned findings are indicative of a positive insect-related effect on the gut microbiota of the birds.

At the phylum level, the female broilers fed the 10 and 15% levels of TM meal inclusion in the current research displayed a decreased abundance of *Firmicutes* phylum and lower *Firmicutes*:*Bacteroidetes* ratios, respectively, than TM5-fed birds. It is well known that bacteria within the *Firmicutes* phylum may significantly influence both feed digestion and host health [40]. Furthermore, greater *Firmicutes*:*Bacteroidetes* ratios are generally associated with bacterial profiles that show a higher capacity of energy harvesting [41]. Based on these considerations, lower insect levels seem to be preferable for better modulation of gut microbiota. However, it is interesting to underline that the relative abundance of *Firmicutes* phylum began to increase from TM10 to TM15 diets, thus partially attenuating these negative findings.

In regards to the genera composition, the female broiler chickens fed TM in the present study showed increased abundances of *Clostridium*, *Sutterella* and *Alistipes* genera in their cecal microbiota, along with a lower percentage of *Ruminococcus* than C-fed birds. As already reported, *Clostridium* and *Ruminococcus* represent two of the main bacterial genera observed in the chicken cecum [25,31,32]. The *Clostridium* genus also encompasses bacteria capable of producing butyric acid [42,43], which has been reported to positively influence the growth performance, intestinal villus structure and control of naturally occurring pathogens, as well as to show remarkable anti-inflammatory properties [44]. In addition, *Ruminococcus* is capable of producing other SCFAs (i.e., acetic and succinic acid) through glucose metabolism and cellulose digestion [45]. SCFA production is of vital importance for intestinal health, since they are a remarkable source of energy for enterocytes [46] and can suppress gut pathogens [47]. With regards to the other differentially abundant OTUs identified in the present study, the *Alistipes* genus has been reported to be a bile-resistant organism with the ability to produce fibrinolyisin, digest gelatin and ferment carbohydrate in order to produce acetic acid, thus being considered gut beneficial bacteria [48]. Furthermore, the *Sutterella* genus—which is generally identified in pigeon “milk”—has been reported to be a potential probiotic capable of improving the growth rate and the feed conversion ratio of chickens [49]. However, the lack of information about the specific role of *Alistipes* and *Sutterella* in poultry microbiota underlines the importance of future, better characterization of these bacteria.

The increase in *Clostridium*, *Alistipes* and *Sutterella* taxa suggests that dietary TM meal inclusion may exert a positive influence on the cecal microbiota of the birds, with the only potential negative finding being represented by the reduction of *Ruminococcus*. Despite a clear cause–effect relationship between the diversity and composition of cecal microbiota and bird performance having not yet been confirmed, the gut microbiota findings need to be contextualized with those related to animal performance. Biasato et al. [10] previously reported that increasing levels of dietary TM meal inclusion improved the body weight and feed intake of birds without impairing overall feed efficiency. Therefore, the above-mentioned, potential negative gut microbiota findings in terms of both the phyla and the genera composition related to the TM meal utilization did not influence the overall growth performance of the animals. This scenario may also be explained by the unaffected gut morphology observed in the same birds [10]. Indeed, gut health is a complex, multi-factorial concept, to which the assessment different aspects need to be taken into account.

### 4.2. Intestinal Mucin Composition

Similar to gut microbiota characterization, the current research is the first one investigating the gut mucin composition of broiler chickens fed insect-based diets.

Greater mucin staining intensity was observed in the intestinal villi of the broiler chickens fed the 5% level of insect meal inclusion in the present study when compared to the 10 and 15%, with the birds fed the 10% level of TM meal inclusion also showing lower mucin staining intensity than those fed the basal diet. Forder et al. [50] previously demonstrated that bacteria are capable of influencing mucin production. The authors pointed out that some bacteria possess mucin-specific glycosulfatases that can cleave sulfate from its linkage to mucin sugars [5]. As colonization becomes greater, the chicken gut needs for greater protection against mucus degradation and an increase in sialomucins production may be observed [50]. As already mentioned before, the broiler chickens in the present study showed a high abundance of the *Helicobacter* genus in their cecal microbiota (with the maximum percentages observed in the birds fed the 10% inclusion level of TM meal), thus suggesting a direct relationship between reduced gut mucin production and the identified bacterial population. Another important aspect to underline is that mucins are involved in the digestion and absorption of nutrients, represent a substrate for the resident flora and facilitate the removal of pathogenic bacteria by aggregation [3]. As a consequence, independently to the gut microbiota findings, the utilization of TM meal at low inclusion rates (i.e., 5%) may be preferable to preserve the positive properties of the mucins.

Independently to TM meal utilization, the intestinal crypts of the broiler chickens in the current research showed greater neutral mucin staining intensity than acidic sialylated and sulfated. The production of neutral mucins represents a protective mechanism against invasion by pathogenic bacteria [51] and can increase intestinal maturity in order to facilitate the breakdown of complex carbohydrates [50], thus representing a positive finding. The cecal intestinal crypts of the broiler chickens fed either the basal or the TM-based diets also showed lower mucin staining intensity in comparison with the other gut segments. Tsirtsikos et al. [21] and Biasato et al. [9] recently observed the same scenario in poultry, with the latter authors attributing this result to the different anatomy and physiology of the cecum [9].

The intestinal villi of the broiler chickens in the present study showed greater mucin staining intensity in the ileum compared with the other gut segments, thus representing a TM meal utilization-independent observation. This finding confirms previous results in chickens, where a progressive, distal increase in the density of goblet cells along the duodenal–ileal axis was highlighted [9,50,52]. The distal ileum has previously been reported to be a potential preferred region for bacterial colonization [50]. Therefore, the microbial dynamics potentially occurring in the ileum may explain the need for higher protection and subsequent greater mucin production.

The intestinal crypts of the birds fed either the C or the TM-based diets in the current research revealed greater mucin staining intensity in the base fragment when compared to the others. This finding is in agreement with the previous studies carried out in chickens, where the decreased mucin stain in the crypt tip has been suggested to depend on the process of proliferation and maturation of the goblet cells [9,21,53]. Higher mucin staining intensity was also observed in the base fragment of the intestinal villi when compared to the others, as analogously observed by Biasato et al. [9]. This result was attributed to the physiological proliferation process of the goblet cells occurring in the villus compartment [9].

## 5. Conclusions

In conclusion, dietary TM meal inclusion was capable of modulating both the gut microbiota and the mucin composition of broiler chickens. In particular, insect meal utilization (especially the 10–15% inclusion levels) may negatively influence either cecal microbiota or intestinal mucin dynamics in terms of the reduction in *Firmicutes* phylum and *Firmicutes*/*Bacteroidetes* ratios and decrease in the mucin synthesis, thus suggesting that lower inclusion levels (i.e., 5%) may be preferable. The SCFA production may have a key role in the gut microbiota dynamics of insect-fed broiler chickens, but further research also adopting an “–omic” approach (i.e., metagenomics and meta-metabolomics) is highly recommended to confirm this hypothesis. Finally, despite the observed potential negative effects, the identification of a physiological cecal community and gut mucin dynamics in all the animals (observed independently of TM meal utilization) represents a promising result in terms of gut health preservation.

## Figures and Tables

**Table 1:** Effects of diet, mucin type, gut segment and crypt-villus fragment on mucin staining intensity in broiler chickens.

Factor	d.f. ^6^	Chi-Square	P ^7^
Crypts			
Diet ^1^	3	3.736	0.291
Mucin type ^2^	2	10.084	0.006
Gut segment ^3^	3	216.132	<0.001
Fragment ^4^	2	112.127	<0.001
Villi			
Diet	3	12.569	0.006
Mucin type	2	0.762	0.683
Gut segment ^5^	2	140.155	<0.001
Fragment	2	6.561	0.038

^1^ Four dietary treatments: C = control; TM5 = 5% inclusion level of *Tenebrio molitor*; TM10 = 10% inclusion level of *Tenebrio molitor*; TM15 = 15% inclusion level of *Tenebrio molitor*. ^2^ Three types: neutral, acidic sialylated and acidic sulfated mucins. ^3^ Four gut segments: duodenum, jejunum, ileum and cecum. ^4^ Three fragments: base, midsection and tip. ^5^ Three gut segments: duodenum, jejunum and ileum. ^6^ Degrees of freedom. ^7^ Statistical significance: *p* < 0.05.

**Table 2:** Mucin staining intensity in the intestinal crypts of the broiler chickens depending on diet, mucin type, gut segment and crypt-villus fragment.

Gut Mucosal Element	Predictor	Predictor Factors	Mucin Staining Intensity ^1,2^
Crypts	Diet	C	1.23 ± 0.03
		TM5	1.26 ± 0.03
		TM10	1.31 ± 0.03
		TM15	1.26 ± 0.03
	Mucin type	Neutral	1.33 ± 0.03 ^A^
		Acidic sialylated	1.23 ± 0.02 ^B^
		Acidic sulfated	1.24 ± 0.02 ^B^
	Gut segment	Duodenum	1.18 ± 0.03 ^C^
		Jejunum	1.40 ± 0.03 ^B^
		Ileum	1.55 ± 0.03 ^A^
		Cecum	1.00 ± 0.02 ^D^
	Fragment	Base	1.49 ± 0.03 ^A^
		Midsection	1.18 ± 0.02 ^B^
		Tip	1.15 ± 0.02 ^B^
Villi	Diet	C	1.82 ± 0.04 ^AB^
		TM5	1.92 ± 0.05 ^A^
		TM10	1.70 ± 0.04 ^C^
		TM15	1.77 ± 0.04 ^BC^
	Mucin type	Neutral	1.83 ± 0.04
		Acidic sialylated	1.79 ± 0.04
		Acidic sulfated	1.78 ± 0.04
	Gut segment	Duodenum	1.50 ± 0.03 ^B^
		Jejunum	1.83 ± 0.04 ^A^
		Ileum	2.13 ± 0.04 ^A^
	Fragment	Base	1.87 ± 0.04 ^a^
		Midsection	1.79 ± 0.04 ^ab^
		Tip	1.73 ± 0.04 ^b^

^1^ Data are represented as the mean of counts ± SEM. ^2^ Means with different superscript letters (a, b or A, B, C, D) within the same column per predictor (i.e., diet, mucin type, gut segment or fragment) differ significantly (*p* < 0.05 or *p* < 0.01, respectively). C = control; TM5 = 5% inclusion level of *Tenebrio molitor*; TM10 = 10% inclusion level of *Tenebrio molitor*; TM15 = 15% inclusion level of *Tenebrio molitor*.

## Supplementary Materials

The following are available online at https://www.mdpi.com/2076-2615/9/5/213/s1. Table S1: Ingredients and chemical composition of the experimental diets (the same for both experimental trials). Mineral-vitamin premix (Final B Prisma, IZA SRL, Forlì, Italy), given values are supplied per kg of diet: 2.500.000 IU of vitamin A; 1.000.000 IU of vitamin D3; 7.000 IU of vitamin E; 700 mg of vitamin K; 400 mg of vitamin B1; 800 mg of vitamin B2; 400 mg of vitamin B6; 4 mg of vitamin B12; 30 mg of biotin; 3.111 mg of Ca pantothenate acid; 100 mg of folic acid; 15.000 mg of vitamin C; 5.600 mg of vitamin B3; 10.500 mg of Zn, 10.920 mg of Fe; 9.960 mg of Mn; 3.850 mg of Cu; 137 mg of I; 70 mg of Se. AMEn = apparent metabolizable energy; CP = crude protein; EE = ether extract; NDF = neutral detergent fiber; ADF = acid detergent fiber; C = control; TM5 = 5% inclusion level of *Tenebrio molitor*; TM10 = 10% inclusion level of *Tenebrio molitor*; TM15 = 15% inclusion level of *Tenebrio molitor*. Table S2: Good’s coverage and α-diversity measures of cecal microbiota of female broiler chickens fed control (C), 5% (TM5), 10% (TM10) and 15% (TM15) inclusion level of *Tenebrio molitor* meal diets. Description column indicates the 5 replicate pens of control (C_1, C_2, C_3, C_4 and C_5), 5% (TM5_1, TM5_2, TM5_3, TM5_4 and TM5_5), 10% (TM10_1, TM10_2, TM10_3, TM10_4 and TM10_5) and 15% inclusion level of *Tenebrio molitor* meal (TM15_1, TM15_2, TM15_3, TM15_4 and TM15_5) dietary treatments. Table S3: Relative abundance of the main bacterial phyla and genera of cecal microbiota of broiler chickens fed control (C), 5% (TM5), 10% (TM10) and 15% (TM15) inclusion level of *Tenebrio molitor* meal diets.
